# Holistic management of symptomatic reflux: rising to the challenge of proton pump inhibitor overuse

**DOI:** 10.3399/bjgp22X721157

**Published:** 2022-10-28

**Authors:** Edoardo Savarino, Foteini Anastasiou, Joachim Labenz, A Pali S Hungin, Juan Mendive

**Affiliations:** Department of Surgery, Oncology and Gastroenterology, University of Padua, Padua, Italy.; University of Crete, Heraklion, Greece; Coordinator, 4th Local Primary Care Team (TOMY), Municipality Practice, Academic Practice of Heraklion, Greece.; Department of Internal Medicine, Diakonie Klinikum Jung-Stilling Hospital, Siegen, Germany.; Faculty of Medical Sciences, University of Newcastle, Newcastle upon Tyne, UK.; La Mina Primary Care Academic Centre, Catalan Institute of Health, University of Barcelona, Barcelona, Spain.

## THE ISSUE OF CONTINUED PPI OVERUSE

Proton pump inhibitors (PPIs) are medications that suppress the production of stomach acid.^1^ Introduced to clinical practice over 30 years ago, PPIs gained rapid popularity for the effective treatment of acid-related disorders and are now some of the most prescribed therapeutics worldwide.^2^ PPIs are indicated for conditions such as gastro-oesophageal reflux disease (GORD), peptic ulcer disease, Zollinger–Ellison syndrome, and *Helicobacter pylori* infection, and are also recommended for gastroprotection against non-steroidal anti-inflammatory drugs (NSAIDs) and anti-platelet treatments that can induce pathologies in at-risk patients.^1^

GORD is prevalent in populations adopting a Western-style diet characterised by large and calorie-dense meals, and presents a significant individual, economic, and societal burden.^3^^,^^4^ The 2006 Montreal Definition states that GORD is *‘a condition that develops when the reflux of stomach contents causes troublesome symptoms and/or complications’*.^5^ While widely agreed to be a breakthrough in the classification of symptomatic reflux, the definition is not without limitations and issues arising from its ambiguity have had a significant impact. In providing rationale for acid suppression without investigation of the cause and nature of symptoms,^6^ the definition opened treatment options up to an extremely large patient population. A consequence of this has been an increase in PPI prescribing, particularly for extra-oesophageal conditions potentially associated with GORD.

Long-term PPI therapy has been associated with an increased risk of kidney disease, gastric fundic polyps, enteric infections, and nutritional deficiencies, among other conditions. However, it should be noted that evidence of any association is weak and rigorous evaluation is lacking.^7^ Despite this and guideline recommendations, there is a wealth of evidence demonstrating that long-term PPI use is widespread. One analysis of PPI prescribing trends showed that 37.5% of patients remain on their original treatment course for over a year, contrary to recommendations.^8^ Paucity in structured follow-up following hospital discharge back into primary care has been proposed as one reason for this phenomenon, with one evaluation demonstrating that only 18% of discharge letters reviewed gave the intended duration of PPI therapy, and only 7% a proposed review date.^9^

It has also been shown that many patients continue to take PPIs despite a lack of evidence of reflux disease.^10^ Up to one-third of symptomatic cases falling under the Montreal Definition are refractory to PPI treatment, and whether these cases represent true refractory disease or are indicative of alternative, non-GORD aetiologies is debated. Guidelines state that most cases of uncomplicated GORD should resolve within 4–8 weeks of treatment, after which PPIs should be de-escalated.^11^^,^^12^ Another reason for increased prescribing may be that these patients, who achieve no or only partial benefit from PPIs, may be more likely to undergo dose escalation with protracted use at higher doses. In cases of confirmed refractory GORD, or alternative diagnoses unrelated to acid dyspepsia (for example, Barrett’s oesophagus), longer therapeutic periods are deemed appropriate, but only at the lowest effective dose.^11^^,^^12^

## FACTORS CONTRIBUTING TO PPI OVERUSE

Factors contributing to the inappropriate and/or protracted use of PPIs have arisen from challenges presented to both healthcare professionals (HCPs) and patients.

Speaking to the challenges faced by HCPs, the recommendation by the UK’s National Institute for Health and Care Excellence (NICE) that patients be given information about self-management of GORD and dyspepsia (for example, advice on lifestyle changes)^11^ presents a significant time and resource burden on top of already heavy workloads. Reluctance to follow this guidance is then exacerbated by a lack of specific management tools in the absence of specialist allied services. Last, while recognising the need to avoid ‘unnecessary’ long-term treatment, HCPs are also caught in the dilemma of wanting not only to ensure adequate symptom control, but also avoid repeated consultations due to persisting symptoms.^13^

HCPs are also often faced with a strong patient expectation for a pharmaceutical solution for their symptoms; indeed, in one study, physicians expressed the opinion that the *‘pressure to prescribe PPIs* [as exerted by the patient] *was outweighed by the pressure not to prescribe’*.^14^ And yet, in another study, approximately half of patients reported concerns with being on a PPI in the long term, and a third indicated that they wanted to stop their PPI,^15^ demonstrating a clear need for continued patient–physician communication beyond initial prescription. For those patients who go on to derive benefit from PPIs, the impetus to address dietary factors or consider other therapeutic options is removed so long as symptom relief is maintained.^13^ In those who do not benefit, we propose that a gap in understanding of the definition of ‘refractory GORD’, coupled with the range of other potential diagnoses, favours protracted treatment periods and/or PPI dose escalation. This may also delay the decision to investigate other diagnostic and therapeutic options. Limited evidence on the efficacy of alternative therapies when administered alone, including alginates and antacids (which offer symptomatic relief), may compound this issue further by instilling greater HCP trust in the more extensively evaluated PPIs.

HCPs may also be reluctant to embark on de-escalation in cases where they were not the original prescriber, and so lack the full clinical picture of why PPIs were originally recommended. This is a common occurrence on discharge from secondary care, with one audit of 36 primary care practices in Germany indicating that 58% of patients were prescribed PPIs without a clear indication in hospital, leaving the primary care HCP with the arduous task of supporting discontinuation.^16^ Deprescribing in an increasing population also presents a considerable time and resource burden, with efforts including lifestyle counselling and education often requiring participation from several allied HCPs at potentially substantial cost.^1^ However, it should be noted that a 1-year review of a nurse-led PPI deprescribing support programme demonstrated that increased costs associated with implementation were offset by a cost saving of £31 716 on the annual prescribing budget for the practices in the study brought about by a 49% reduction in PPI prescriptions.^2^

Though comprehensive on PPI prescribing, available guidelines lack clarity on how to support patients through discontinuation where no benefit is observed, or once symptom resolution is achieved. Advice pertaining to safe and effective de-escalation while maintaining symptom control is particularly underrepresented. The increase of gastric acid secretion above pre-treatment levels in incidences of rebound acid hypersecretion (RAHS) may motivate patients to restart PPI therapy, which must then be carefully managed by the physician.^17^ This is especially challenging in supporting discontinuation following long-term use since the duration of PPI use is a significant predictive factor for symptom recurrence on step-down.^18^

## THE PATIENT PERSPECTIVE

We propose that patient reluctance to question their need for PPI therapy is likely influenced by the efficacy of PPIs combined with their generally tolerable short-term safety profile. Many see the symptom resolution by their PPIs as a simple, immediate solution, as opposed to the long and difficult process of lifestyle modification. The desire for *‘something on prescription’* may also be a reason to suggest that a placebo effect may be at play, at least in part, for the symptom reduction experienced during a short PPI trial. As such, we believe that, with adequate deprescribing support, a short treatment window with careful observation can lead to lasting lifestyle changes initiated by this effect.

In our opinion, there exists a lack of understanding about the mechanism of action among patients, with many believing that PPIs *‘form a protective barrier for the stomach’*. While PPIs are indicated to reduce the risk of gastric pathologies caused by NSAIDs, no such risk exists for most other commonly prescribed therapeutics — and yet there is a perception (among patients and some HCPs) that PPIs are *‘universally gastroprotective’* in polypharmacy. That is, patients may perceive PPIs to be necessary to prevent gastric damage caused by the combined side effects of other medications. This is particularly prevalent in patients with chronic comorbidities on therapeutic regimens amounting to several pills per day. This assertion is incorrect and highlights an opportunity for improved education for HCPs on appropriate prescribing, and for patients, on the limited range of situations where they can expect to derive benefit from PPIs.

Another potential factor influencing the patient is cost. For many seeking symptom relief, PPIs represent a cost-effective solution. PPIs are readily available and inexpensive, or even free on prescription in some settings. Over-the-counter alternatives, which achieve similar or improved luminal threshold pH levels and are efficacious in the treatment of symptomatic GORD, may be purchased by the patient on an as-needed basis. Thus, there may be a financial incentive for the patient to trial PPIs before exploring self-management options, potentially at the expense of adequate and sustained therapeutic relief.

It is imperative to articulate the incentives for de-escalation of PPIs for long-term users who have experienced effective symptom resolution. Sentiments such as, *‘I’ve been on these for 20 years, why should I stop now?’* and, *‘It’s working — so why change it?’* typify the attitudes of patients who see no reason to reassess their therapeutic need, particularly in the face of risk of RAHS. Counselling on PPI limitations, combined with supportive and patient-centric deprescribing practices, including over-the-counter symptom relief for RAHS, could see the return to self-care for these individuals.

Despite barriers to deprescribing, evidence suggests that patients are willing to strive for better outcomes with respect to PPI use. Interviews with patients and HCPs have found that HCPs underestimated patient concerns about the long-term use of PPIs, and uncovered patients’ motivation to work with HCPs to achieve the minimum effective dose through experimentation.^14^ These findings were complemented by another study in which patients were found more willing to adjust their usage when they were actively encouraged to be involved in the medication review process.^19^

## HOW CAN DISCONTINUATION BE BETTER SUPPORTED?

We propose a novel, patient-focused, holistic approach to management throughout the process of PPI prescribing and deprescribing, where the key is symptom control. This includes:

### Emphasis on treating the individual patient holistically, rather than treating the acid-based component of the condition

Each patient has individual requirements, symptom contributors, dietary attitudes and habits, and therefore each case of symptomatic reflux requires a personalised approach. Ideally, physicians should be supported by allied HCP colleagues in counselling behaviour change, setting out the ‘rules of engagement’ early in the prescribing conversation, that is, PPIs should be trialled in a window of opportunity for patients to address underlying causes with support.

### An educational programme, targeting both patients and treating physicians, to reposition PPIs as a temporary solution for symptom control

A global educational campaign is needed, emphasising the role of PPIs in symptomatic reflux as a temporary measure to be taken while underlying factors are addressed. This should include education of both patients and HCPs on appropriate deprescribing and self-management through alternatives such as alginates, to result in a sustainable reduction in PPI use worldwide.

The utility of an informative and motivational leaflet was recently tested in a randomised controlled trial including 140 Lebanese patients. At the 6-month follow-up, significantly more study participants had talked to their treating physician and/or stepped down or come off their PPI in the intervention vs. control group (both *P*<0.0001).^20^ This study is a fine example of the impact that a low-intensity, low-cost educational resource can have, and a similar approach is currently being tested in French cohorts.^21^

Where possible, nurse-led programmes should be implemented to help drive patient empowerment while reducing the potential safety and financial implications of long-term PPI treatment. This complements evidence suggesting that step-down practices are more likely to be successful and sustainable with a combined approach of education and symptom support.^19^ In a UK initiative supported by rescue therapy for rebound symptoms, 75% of patients were able to step down or off their PPI across nine primary care centres, with an annual practice cost saving of £31 716, demonstrating the potential of such an approach.^2^ In a similar study, during which patients in rural Scotland were supported by a specialist nurse advisor, 83% were able to step down or off their PPI, with an annual practice cost saving of £3180.^22^

Coaching should not be limited to patients — treating physicians should also receive targeted training on how to recognise and reduce inappropriate prescribing of PPIs. Impressive results have been demonstrated with educational programmes in dementia, leading to reduced antipsychotic prescribing and improved staff knowledge and attitudes,^23^ and a similar initiative should be applied in GORD. Multifaceted strategies including educational YouTube videos and Twitter campaigns would further maximise the reach and uptake of guidelines.^24^

### A clear and active role for the patient in bringing about their transition to self-care

Evidence indicates that improved outcomes are achieved when patients are brought into the deprescription decision-making process as a partner.^19^ A strong doctor–patient relationship is also key to ensuring medication compliance,^25^ and should support a stepwise reduction in PPI use. Each patient should undergo a 4–8-week review (in line with current guidelines) where they will be expected to contribute to the evaluation.

### Self-directed over-the-counter short-term symptom relief for rebound symptoms

UK NICE guidelines state that a return to self-care can be achieved with a stepwise reduction in PPI medication by means of antacids and/or alginates to manage intermittent symptoms.^11^ These easily accessible options can provide fast symptomatic relief and reduce the risk of relapse.

### Improved guidelines for deprescribing PPIs to bring them in line with prescribing guidelines

We believe that the Canadian deprescribing guidelines are the most comprehensive available and suggest that those devising or restructuring guidelines to add clarity on deprescribing look to these as a model.^26^ The use of case studies alongside deprescribing guidelines would be useful for HCP and patient understanding.

Box 1 summarises the key points of this article.

**Box 1.** Key points

Proton pump inhibitors (PPIs) are some of the most prescribed medications worldwide. In cases of uncomplicated gastro-oesophageal reflux disease (GORD), a 4–8 week treatment window is usually sufficient for symptom resolution. However, inappropriate and/or sustained use of PPIs where there is no clear indication, or when no benefit is observed, is common. There is a need for discussion of factors underlying inappropriate PPI use, with a view to better supporting primary care physicians through sustainable deprescribing practices. We, a group of international experts in gastroenterology and primary care, call for a more holistic approach to symptom management on a per-patient basis, over a reductionist view of the acid-based condition alone. PPI prescribing and deprescribing should be supported by behavioural change, improved education, and symptom relief while the patient returns to self-care. Research is needed to support the development of guidelines and educational resources.
